# Cardiac myocyte miR-29 promotes pathological remodeling of the heart by activating Wnt signaling

**DOI:** 10.1038/s41467-017-01737-4

**Published:** 2017-11-20

**Authors:** Yassine Sassi, Petros Avramopoulos, Deepak Ramanujam, Laurenz Grüter, Stanislas Werfel, Simon Giosele, Andreas-David Brunner, Dena Esfandyari, Aikaterini S. Papadopoulou, Bart De Strooper, Norbert Hübner, Regalla Kumarswamy, Thomas Thum, Xiaoke Yin, Manuel Mayr, Bernhard Laggerbauer, Stefan Engelhardt

**Affiliations:** 1 Institute of Pharmacology and Toxicology, Technical University Munich (TUM), 80802 Munich, Germany; 2 DZHK (German Center for Cardiovascular Research), partner site Munich Heart Alliance, 80802 Munich, Germany; 3 VIB Center for the Biology of Disease, VIB, 3000 Leuven, Belgium; 4 Center for Human Genetics and Leuven Institute for Neurodegenerative Disorders (LIND), KU Leuven and Universitaire Ziekenhuizen, 3000 Leuven, Belgium; 5 Cardiovascular and Metabolic Sciences, Max-Delbrüeck-Center for Molecular Medicine in the Helmholtz Association (MDC), 13125 Berlin, Germany; 6 DZHK (German Center for Cardiovascular Research), Partner Site Berlin, 10115 Berlin, Germany; 7 Charité-Universitätsmedizin, 10117 Berlin, Germany; 8 Institute of Molecular and Translational Therapeutic Strategies (IMTTS), Hannover Medical School, 30625 Hannover, Germany; 9 King’s British Heart Foundation Centre, King’s College London, SE5 9NU London, UK; 10 Present Address: Mount Sinai, Cardiovascular Research Center, Icahn School of Medicine at Mount Sinai, New York, NY 10029 USA

## Abstract

Chronic cardiac stress induces pathologic hypertrophy and fibrosis of the myocardium. The microRNA-29 (miR-29) family has been found to prevent excess collagen expression in various organs, particularly through its function in fibroblasts. Here, we show that miR-29 promotes pathologic hypertrophy of cardiac myocytes and overall cardiac dysfunction. In a mouse model of cardiac pressure overload, global genetic deletion of miR-29 or antimiR-29 infusion prevents cardiac hypertrophy and fibrosis and improves cardiac function. Targeted deletion of miR-29 in cardiac myocytes in vivo also prevents cardiac hypertrophy and fibrosis, indicating that the function of miR-29 in cardiac myocytes dominates over that in non-myocyte cell types. Mechanistically, we found cardiac myocyte miR-29 to de-repress Wnt signaling by directly targeting four pathway factors. Our data suggests that, cell- or tissue-specific antimiR-29 delivery may have therapeutic value for pathological cardiac remodeling and fibrosis.

## Abstract (web summary)

MicroRNA-29 is known to reduce collagen production in fibroblasts thereby inhibiting fibrosis in various organs. Here, Sassi et al. show that miR-29 can also enhance fibrotic signalling and pathological hypertrophy of the heart through its action in cardiomyocytes.

## Introduction

MicroRNAs (miRNAs) are short, non-coding RNA molecules that regulate gene expression at the post-transcriptional level^1^, and serious estimates are that the majority of genes and cellular processes are controlled by miRNAs or other non-coding RNA molecules^2–4^. miRNAs have likewise been implicated in many diseases^5^, including those of the cardiovascular system^6^. Of particular interest in this respect is cardiac remodeling, a response of the myocardium to chronic cardiac stress conditions, such as aortic stenosis, that is marked by cardiac hypertrophy and fibrosis. Hypertrophy and fibrosis are tightly interwoven, and mutually trigger each other^7^. Prominent miRNAs with a documented role in cardiac myocyte hypertrophy include miR-208, miR-133 and miR-212/132^8–10^, whereas a role in fibrosis has been demonstrated for miR-21, miR-30, miR-133, and miR-29^11–15^. In this regard, miR-29 has been reported to be downregulated in several fibrosis-related diseases in rodents as well as in humans, and has been shown to target mRNAs that encode fibrosis-promoting proteins in different cell types/organs^16–21^. In apparent agreement with this, experimental elevation of miR-29 was found to repress collagen transcripts in cultured cardiac fibroblasts^13^. Altogether, these findings supported the concept that enhancing miR-29 would be a promising anti-fibrotic therapy for lung, liver, kidney and heart diseases. At the same time, there are exciting reports about the ability of specific miR-29 inhibitors (antimiRs) to prevent development or progression of abdominal aortic aneurysms^22–24^. AntimiR-29 has proven even more effective in this context than antimiRs against another promising candidate, miR-195^25^.

Remarkably, the miR-29 family (i.e., miR-29a, b, and c) had also emerged as a potent inducer of cardiac myocyte (CM) hypertrophy from a phenotypic screen^26^. As this might have consequences for the therapeutic use of miR-29, we set out to elucidate the role of miR-29 in myocardial remodeling, and manipulated miR-29 in primary CMs, as well as in vivo. Here, we show that miR-29 promotes hypertrophic growth of cardiac myocytes in vivo, together with an increase, rather than a reduction, of cardiac fibrosis. In support of this finding, pharmacological inhibition or genetic deletion of miR-29 prevented cardiac hypertrophy and fibrosis in mice. miR-29 was further characterized as a regulator of canonical and non-canonical Wnt signaling in cardiac myocytes, thereby eliciting hypertrophy of these cells and the secretion of profibrotic proteins that signal towards cardiac fibroblasts (CF). This indicates a unique hierarchy of miR-29 activities in cardiac cell types with respect to cardiac remodeling, with effects of miR-29 in CM dominating over its function in CF.

## Results

### Genetic deficiency of miR-29 prevents cardiac remodeling

**Fig. 1:**
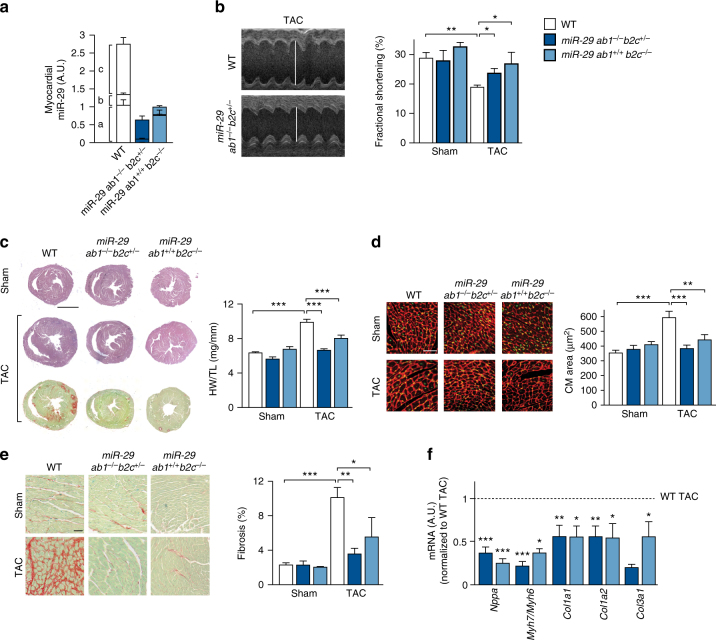
Genetic deletion of miR-29 in a mouse model for left ventricular pressure overload. **a** Expression of miR-29 family members in left myocardium from wildtype (WT), *miR-29 ab1* ^*−/−*^ *b2c* ^*+/−*^ and *miR-29 ab1* ^*+/+*^ *b2c* ^*−/−*^ mice; *n* = 4–6 mice/group. **b** Echocardiographic analysis of fractional shortening as a measure of left ventricular function; *n* = 4–10 mice/group. A Student’s *t*-test was used to calculate the *P* values. **c** (Left) Representative stainings of myocardial tissue from WT or knockout mice (tissue fixation 21 days after sham surgery or transverse aortic constriction, TAC) by hematoxylin/eosin and Sirius Red/Fast Green stainings. Scale bar: 2 mm. (Right) Ratio between heart weight and tibia length (HW/TL) as a measure of cardiac hypertrophy; *n* = 6–14 mice/group. *P* values were determined by two-way ANOVA followed by Bonferroni’s post hoc test. **d** (Left) Representative wheat germ agglutinin (WGA)-staining of midventricular sections to assess hypertrophy of cardiac myocytes. Scale bar: 50 µm. (Right) Quantitative analysis; *n* = 5–8 mice/group. *P* values were calculated using two-way ANOVA followed by Bonferroni’s post hoc test. **e** (Left) Representative image sections from Sirius Red/Fast Green-stained myocardium of the indicated groups and (Right) quantitative analysis of fibrosis; *n* = 3–11 mice/group. *P* values were determined by two-way ANOVA followed by Bonferroni’s post hoc test. **f** Real-time PCR quantification of markers for cardiac remodeling in left ventricular tissue from WT, *miR-29 ab1* ^*−/−*^ *b2c* ^*+/−*^ and *miR-29 ab1* ^*+/+*^ *b2c* ^*−/−*^ mice. Collagen mRNAs and the following markers of cardiac myocyte hypertrophy were assessed: *Nppa*, atrial natriuretic peptide; *Myh7/Myh6*, ratio of mRNAs encoding β- and α-myosin heavy chain. Tissues were collected 21 days after TAC surgery; data are from 4 to 9 independent experiments, with 2 replicates each. WT TAC means were compared to that of *miR-29 ab1* ^*−/−*^ *b2c* ^*+/−*^ and *miR-29 ab1* ^*+/+*^ *b2c* ^*−/−*^ mice by a one-way ANOVA followed by a Bonferroni’s post hoc test. *P < 0.05, **P < 0.01, ***P < 0.001 for all panels. All quantitative data are reported as means ± SEM

Starting from the observation that raising miR-29 levels and activity by synthetic miR-29 molecules induced hypertrophy of primary cardiac myocytes in vitro (Supplementary Fig. 1), we asked whether inhibition or reduction of this miRNA was beneficial in cardiac disease models in vivo. We did not obtain mice with complete genetic loss of both miR-29 clusters (*miR-29, a/b1, and b2/c*), which is in good agreement with a recent report^27^, according to which the offspring ratio was 1%, with those born dying within few weeks. Therefore, we used mouse lines with partial deficiency of miR-29 variants, that is, mice with *miR-29 ab1* ^*−/−*^ *b2c* ^*+/−*^ or *ab1* ^*+/+*^ *b2c* ^*−/−*^ genotype. Again consistent with other studies^27–29^, mice with triple-allelic deletion of miR-29 show some growth retardation (Supplementary Fig. 2a) and increased mortality, whereas mice with *miR-29 ab1* ^*+/+*^ *b2c* ^*−/−*^ genotype had normal life spans and did neither show any phenotypical effects as, for instance, in body weight nor was there evidence for the induction of fibrosis in other organs (Supplementary Fig. 2b). Cardiac miR-29 levels (miR-29 referring here and in the following to the respective 3p strands, as we found the 5p strands to be hardly expressed) in *miR-29 ab1* ^*−/−*^ *b2c* ^*+/−*^ or *ab1* ^*+/+*^ *b2c* ^*−/−*^ mice were reduced by 80% or 65%, respectively (Fig. 1a), but–under basal conditions–this deficiency did not elicit a cardiac phenotype (Fig. 1b–e). We then proceeded to test these mice in a disease model for left ventricular pressure overload, induced by transverse aortic constriction (TAC). TAC-treated *miR-29 ab1* ^*−/−*^ *b2c* ^*+/−*^ or *ab1* ^*+/+*^ *b2c* ^*−/−*^ mice showed a reduction of miR-29 by 60% or 40%, respectively (Supplementary Fig. 3a). Importantly, both mouse lines were protected from TAC-induced cardiac hypertrophy and functional impairment, showing better heart function than wildtype (Fig. 1b and Supplementary Table 1), reduced myocardial (i.e., heart weight) and cardiac myocyte hypertrophy (Fig. 1c, d) and lower levels of hypertrophy-associated marker genes *Nppa* and *Myh7 (*in relation to *Myh6*) (Fig. 1f). Remarkably, also myocardial fibrosis was reduced in these miR-29-deficient mice, as determined by Sirius Red staining of left ventricular sections (Fig. 1c, e) and by collagen expression (Fig. 1f). Comparably minor effects were seen in Sham-treated animals, where only two genes were modestly deregulated (i.e., 2-fold for *Col1a1*, 0.5-fold for *Col3a1* in *miR-29 ab1* ^*−/−*^ *b2c* ^*+/−*^ vs. WT) and matrix staining with Sirius Red was found to be unchanged (Fig. 1e).

### AntimiR-29 protects against cardiac hypertrophy and fibrosis

**Fig. 2:**
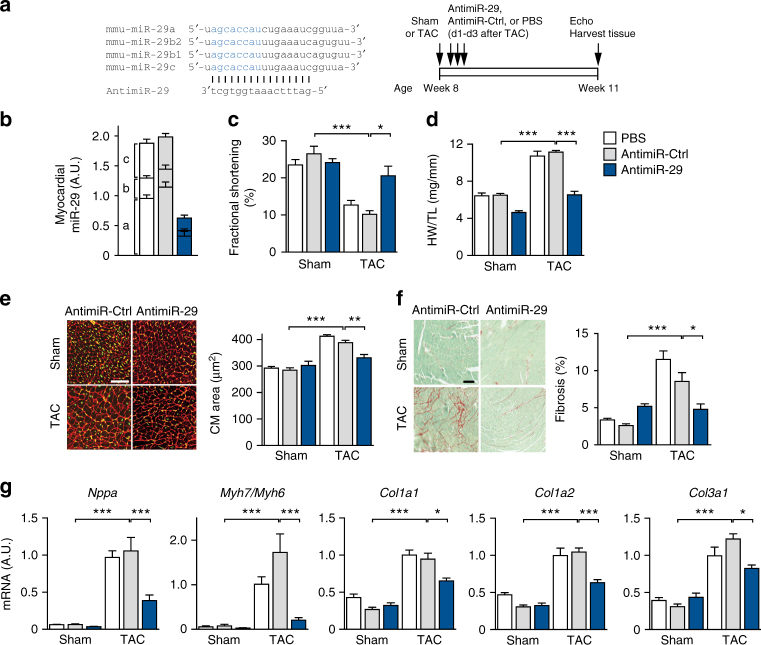
Pharmacological inhibition of miR-29 prevents cardiac remodeling and dysfunction. **a** (Left) Design of the miR-29 family inhibitor (antimiR-29). Sequences of miR-29a, b1, b2 and c display a high degree of sequence similarity with identical seed regions (depicted in blue), thus permitting the design of a single antimiR molecule. (Right) Design of the study. **b** Cardiac expression of miR-29 family members in mice, determined weeks after the first injection of antimiR-29, a control molecule (antimiR-Ctrl) or PBS. **c** Echocardiographic analysis of left ventricular fractional shortening in sham-/TAC-operated mice 3 weeks after injection with antimiR-29/-ctrl or PBS (determined by echocardiography), indicating reduced TAC effects in the antimiR-29-treated group. **d** Heart weight-to-tibia length ratio and **e** WGA staining of left ventricular tissue from mice described in **c** for determination of cardiac and CM hypertrophy. **f** (Left) Representative left ventricular sections stained with Sirius Red/Fast Green of the indicated treatment groups and (right) quantification of interstitial fibrosis. **g** Quantitative real-time PCR analysis of molecular markers for cardiac myocyte hypertrophy (*Nppa*, *Myh7/Myh6*) and of fibrosis-associated collagens. All scale bars: 50 µm. All quantifications derive from *n* = 5–14 mice/group, PCR performed with 2 replicates each. All quantitative data in panels **c**–**g** are reported as means ± SEM. *P < 0.05, **P < 0.01, ***P < 0.001 determined by two-way ANOVA followed by Bonferroni’s post hoc test

To exclude that compensatory mechanisms in *miR-29 ab1* ^*−/−*^ *b2c* ^*+/−*^ or *miR-29 ab1* ^*+/+*^ *b2c* ^*−/−*^ mice had obscured additional effects, we next sought to inhibit miR-29 acutely in the adult animal. Three consecutive injections of a chemically modified antisense oligonucleotide, designed to target all members of the mouse miR-29 family (antimiR-29), lead to a 70% reduction of myocardial miR-29 under basal conditions (Fig. 2a, b) and in the TAC model (Supplementary Fig. 3b). This relative reduction was observed in cardiac myocytes as well as in cardiac fibroblasts (Supplementary Fig. 3c). Consistent with our findings in miR-29-deficient mice, infusion of antimiR-29 protected from cardiac dysfunction, cardiac hypertrophy at the tissue and cellular level, and from fibrosis (Figs. 2c–f and Supplementary Table 2). Likewise, the corresponding gene expression signatures revealed a decrease of markers for hypertrophy (*Nppa, Myh7*) and fibrosis (*Col1a1, Col1a2, Col3a1*) (Fig. 2g). One study reported increased perivascular fibrosis upon application of a miR-29b inhibitor, without affecting cardiac hypertrophy^30^. This could be due to different oligonucleotide chemistries or the specific targeting of variant miR-29b which, due to nuclear localization^31,32^, may have functions other than, or in addition to, variants a and c. Because of these unresolved discrepancies, and in view of the therapeutic effects observed thus far, we chose to analyze in detail the expression of miR-29 variants in healthy and diseased myocardium.

### The miR-29 family is dynamically regulated

**Fig. 3:**
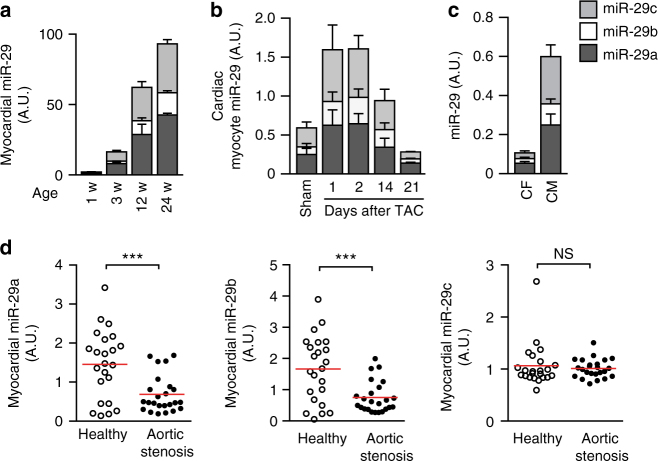
Expression of miR-29 members in cardiac cells and their deregulation in disease. **a**–**d** Individual quantifications (by qPCR) of miR-29 variants a, b and c in primary cardiac cells or in left ventricular myocardium. **a** Age-dependent cardiac expression of miR-29 variants in mice (w: week); *n* = 4–5 mice per group. **b** Endogenous levels of miR-29 family members in cardiac myocytes from mice 21 days after sham treatment or, for the TAC group, at denoted time points; *n* = 3–6 mice per group. **c** Endogenous levels of miR-29 family members in CM and CF freshly isolated from adult mouse myocardium; *n* = 12–13 independent cell isolations. **d** Quantification of miR-29 variants in human left ventricular myocardium from 23 healthy individuals or from 24 patients with aortic valve stenosis. All quantitative data are reported as means ± SEM. ***P < 0.001 calculated using Student’s *t*-test

By quantitative real-time PCR analysis, we found myocardial levels of each miR-29 variant strongly increased with age (Fig. 3a). This is consistent with previous reports^22,33^ and interesting with regard to a proposed function of miR-29 in body growth control^33^. Most remarkable with respect to disease, however, are the temporal changes in miR-29 expression that occur in mice subjected to TAC. This model of pressure overload lead to a prominent increase of cardiac myocyte miR-29 in the first 48 h after surgery, followed by downregulation in the late phase (Fig. 3b). Upon isolation of myocytes and fibroblasts from myocardium of these mice, total as well as individual levels of miR-29 variants were substantially (×6) higher in CM compared to CF (Fig. 3c). This finding is remarkable, given previously reported, higher levels in CF than in CM^13^. We identified a possible explanation for this discrepancy by quantifying miR-29 in CF before and after cultivation: within one week of cultivation, an abnormal and drastic upregulation of miR-29 occurred in primary CF from neonatal rat as well as from adult mouse (Supplementary Fig. 3d, e). Besides this cell culture-related phenomenon in CF, the high endogenous expression of all miR-29 variants in CM suggests a role in this cell type. Interestingly, downregulation of miR-29a and miR-29b in chronic myocardial disease was not only observed in the murine disease model, but also in myocardium from patients with aortic valve stenosis (Fig. 3d). In line with this, a recent HITS-CLIP analysis of Argonaute protein 2 (Ago2)-bound RNAs in human myocardium found miR-29 targets to be enriched in this context^34^. Altogether, these findings prompted us to investigate the role of miR-29 specifically in CMs in adult animals.

### Cardiac myocyte-specific deletion of miR-29 in vivo

**Fig. 4:**
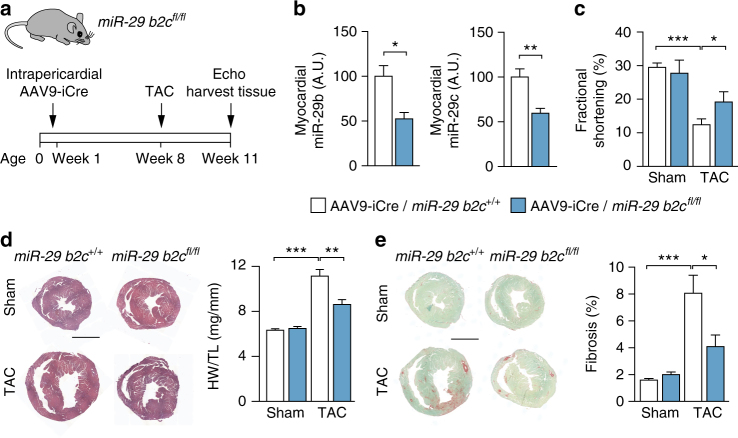
Deletion of miR-29 in cardiac myocytes in vivo protects from cardiac remodeling. Tropism of adeno-associated virus 9 for cardiac myocytes in vivo was employed to deliver improved Cre recombinase (AAV9-iCre) to *miR-29 b2c* ^*fl/fl*^ mice for the deletion of this cluster (with *miR-29 b2c* ^*+/+*^ littermates serving as controls). **a** Design of the study. 5 × 10^11^ viral particles (AAV9-iCre) were delivered to 5 day-old mice via intrapericardial injection. Seven weeks later, mice were subjected to TAC or sham surgery and sacrificed another 3 weeks later (after echocardiographic analysis). **b** Expression of miR-29b and miR-29c in cardiac tissue from mice treated as in **a**; *n* = 4–6 mice per group. **c** Left ventricular fractional shortening as determined by echocardiographic analysis; *n* = 4–8 mice per group. **d** (Left) Representative hematoxylin eosin stainings of myocardial sections; scale bar = 2 mm. (Right) Heart weight-to-tibia length ratio; *n* = 4–11 mice per group. **e** (Left) Representative myocardial sections stained for fibrosis with Sirius Red/Fast Green and (right) quantitative analysis of the results; *n* = 4–9 mice per group; scale bar: 2 mm. All quantitative data are reported as means ± SEM. *P < 0.05, **P < 0.01, ***P < 0.001 as determined by Student’s *t*-test **b** or two-way ANOVA followed by Bonferroni’s post hoc test **c**–**e**

To manipulate miR-29 specifically in CM in vivo, we used adeno-associated virus serotype 9 (AAV9), which targets almost exclusively CMs within the myocardium^15^. In order to genetically inactivate miR-29 specifically in CMs in vivo, we infected mice that carry floxed alleles of the *miR-29b2c* locus (*miR-29 b2c* ^*fl/fl*^) with an AAV9 vector that encodes for improved Cre recombinase (iCre) (Fig. 4a). Treatment with AAV9-iCre reduced myocardial levels of miR-29b and c to approx. 50% (Fig. 4b) and significantly prevented left ventricular dysfunction (Fig. 4c and Supplementary Table 3), myocardial hypertrophy (Fig. 4d) and interstitial fibrosis (Fig. 4e). Altogether, targeted deletion of miR-29 in cardiac myocytes phenocopied pharmacological inhibition or global genetic deletion of miR-29.

As a reciprocal strategy, we adopted our previous approach for AAV9-mediated expression of a microRNA^35^. Upon injection of AAV9-miR-29a (or an AAV9 that encodes the nonrelated *C. elegans* miR-39) into 5 week-old mice (Supplementary Fig. 4a), we observed a 3-fold increase in myocardial miR-29a (compared to control, Supplementary Fig. 4b) that was specific for myocardium and therein for CMs (Supplementary Fig. 4c, d). Under TAC, AAV9-miR-29a mice show enhanced myocardial and CM hypertrophy (Supplementary Fig. 4e, f). In addition, cardiac fibrosis was moderately (yet not significantly) increased without overt cardiac dysfunction (Supplementary Fig. 4g and Supplementary Table 4), which we attribute to the high endogenous miR-29 levels in adult mice (Fig. 3a) and the expected ceiling effect of further experimental elevation. Together, the complementary findings from CM-specific deletion or augmentation of miR-29 suggest a dominant role of miR-29 in CM over that in CF.

### miR-29 de-represses Wnt signaling in cardiac myocytes

**Fig. 5:**
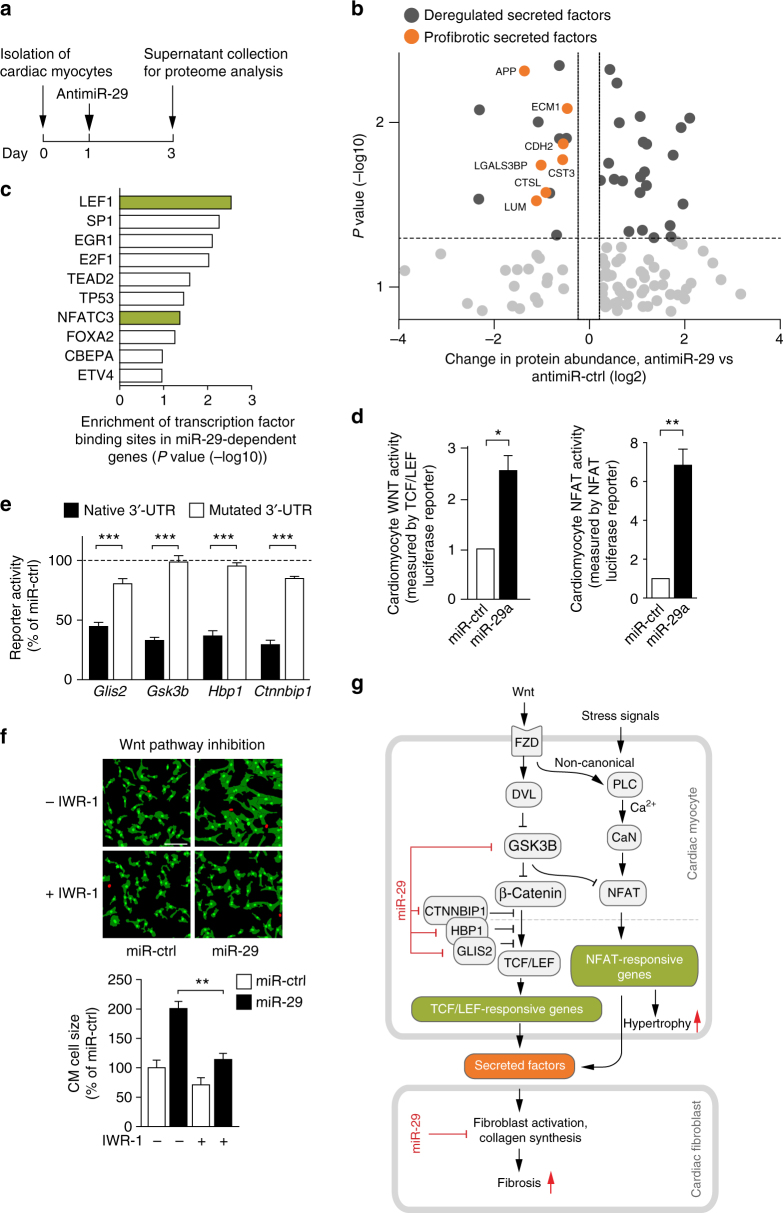
miR-29 targets key components of the Wnt signaling pathway. **a** Design of the study. **b** Volcano-plot of fold changes of individual proteins from NRCM transfected with antimiR-29 or antimiR-ctrl. Dark gray symbols highlight significantly deregulated proteins (*P* < 0.05) and orange symbols those with known or predicted profibrotic function. LUM Lumican, CTSL Cathepsin L, LGALS3BP Galectin 3 Binding Protein, CST3 Cystatin C, CDH2 Cadherin 2, ECM1 Extracellular Matrix Protein 1, APP Amyloid Beta Precursor Protein. **c** Significant GO enrichment of transcription factor binding sites in the deregulated secreted factors. **d** (Left) Wnt activity using a TCF/LEF reporter assay in NRCM 48 h after transfection with miR-29a or miR-ctrl. (Right) NFAT activity in NRCM 48 h after transfection with miR-29a miR-ctrl; 4–5 independent experiments in triplicate. **e** miR-29 directly regulates the *Gsk3β*, *Ctnnbip1*, *Hbp1* and *Glis2* 3´-UTRs. HEK293 cells were transfected with miR-29a or miR-ctrl. Ratiometric analysis of fluorescent emissions from a dual fluorescent reporter carrying the *Gsk3β*, *Ctnnbip1*, *Hbp1* and *Glis2* 3´-UTRs or seed mutants. Data are from 8 independent experiments performed in triplicate. **f** The Wnt-inhibitor IWR-1 (10 μM for 96 h) prevented miR-29-induced cardiac myocyte hypertrophy. (Up) Representative segmentation images of NRCM transfected with either miR-29 or miR-ctrl in the presence or absence of IWR-1 scale bar: 100 μm. NRCM were identified based on α-actinin detection (green) and are assigned green nuclei, whereas non-myocytes were assigned red nuclei. (Down) Quantitative analysis of the results. Data are from four independent experiments performed in triplicate. **g** Proposed mechanism how miR-29 promotes Wnt signaling in CM and signals to fibroblasts. In cardiac myocytes, miR-29 (by targeting *Gsk3b* *Ctnnbip1*, *Hbp1* and *Glis2*) activates the Wnt signaling pathway, as well as NFAT activity. Activation of Wnt and NFAT signaling pathways promotes cardiac myocyte hypertrophy and secretion of profibrotic factors, which act in cardiac fibroblasts. Pharmacological inhibition or genetic deletion of *miR-29* in cardiac myocytes prevents cardiac hypertrophy and fibrosis. All quantitative data are reported as means ± SEM. *P < 0.05, **P < 0.01, ***P < 0.001 as determined by Student’s *t*-test **d**, **e** or two-way ANOVA followed by Bonferroni’s post hoc test **f**

To assess whether miR-29 levels in cardiac myocytes correlate with profibrotic signaling, we analyzed the secretome of primary cardiac myocytes after manipulation of miR-29. CMs were transfected with antimiR-29 or a control antimiR, and proteins from the culture supernatant were isolated and analyzed by mass spectrometry (Fig. 5a). As shown in Fig. 5b, we identified several factors with documented profibrotic function that were reduced in the secretome of antimiR-29-treated CM. Importantly, among the deregulated genes, we found those enriched that have binding sites for TCF/LEF or NFAT (Fig. 5c). The presence of such binding sites marks genes as endpoints of the Wnt/frizzled signaling pathway^36^. Indeed, primary CM after transfection with a miR-29 mimic showed activation of both Wnt ligand-induced and NFAT pathways (Fig. 5d), the latter suggestive of non-canonical Wnt signaling. Since these data suggested that miR-29 may act as a general regulator of Wnt signaling, we further analyzed whether (or which of its) intracellular pathway factors may be under direct control of miR-29. Indeed, four factors of the Wnt pathway contain binding sites for miR-29, namely GSK3B, ICAT/CTNNBIP1, HBP1 and GLIS2. 3′-UTR reporter activity assays (including also control constructs with mutated miR-29 binding sites) identify them as direct targets of miR-29 (Fig. 5e). To assess the functional relevance of the Wnt pathway in mediating the prohypertrophic effects of miR-29, we tested whether a small molecule Wnt pathway inhibitor (IWR-1) would interfere with miR-29-induced cardiac myocyte hypertrophy. Consistent with our earlier findings, elevation of miR-29 levels induced hypertrophy of primary cardiac myocytes, yet IWR-1 largely abrogated the prohypertrophic effect of miR-29 (Fig. 5f). The role of miR-29 as a mediator of NFAT activation became further evident by decreased NFAT-dependent transcriptional activation of Regulator of calcineurin 1 (*Rcan1*) in mice injected with antimiR-29 or miR-29-deficient mice after TAC (Supplementary Fig. 5a), and by the finding that addition of the NFAT inhibitor VIVIT to cultured CM prevents miR-29-induced cardiac myocyte hypertrophy (Supplementary Fig. 5b).

Altogether, these data demonstrate a critical regulation of canonical and non-canonical Wnt signaling in cardiac myocytes through miR-29 (Fig. 5g). Our data are also in agreement with recent HITS-CLIP data^34^ showing that GSK3B and other factors involved in remodeling events in cardiac myocytes are targets of miR-29 in diseased human myocardium. Given that Wnt signaling in CM is increasingly appreciated as a driver not only of cardiac hypertrophy, but also of fibrosis^36–38^, our data suggest a dominant pro-hypertrophic effect in myocardium that is regulated by miR-29.

## Discussion

In this study, we demonstrate a role of the miR-29 family in pathologic hypertrophy of the myocardium and fibrosis. These processes of cardiac remodeling occur in response to chronic cardiac stress, and we show here that inhibition or genetic deficiency of miR-29 protects from cardiac hypertrophy and fibrosis in a mouse model for chronic cardiac pressure overload. In a previous study, we had singled out variants miR-29a and c by phenotypic library screening as potential prohypertrophic microRNAs^26^. The present study substantiates and extends this early observation and identifies cardiac myocyte miR-29 to exert a key role in cardiac remodeling. We show that, in myocardium, miR-29 is prominently expressed in cardiac myocytes (CM) and that CM-specific genetic deficiency of miR-29 in vivo significantly reduced the development of pressure overload-induced cardiac hypertrophy (while, consistently, overexpression in CM in vivo exerted the opposite effect). Intriguingly, analysis of proteins secreted by CM and their validation by 3′ UTR reporter assays suggested that miR-29 exerts these effects, at least in part, by direct regulation of factors of the Wnt signaling pathway.

The Wnt pathway stands out from most other signaling cascades, because its activation occurs by liberation from constitutive inhibition (upon reducing beta-catenin degradation^39^). Our data suggest that the effects of miR-29 on the Wnt cascade occur by repressing several factors that constitute this inhibition (Fig. 5g): (i) GSK3B, the function of which is to commit beta-catenin to degradation and which, in mice transgenic for its hyperactive S9A mutant, protects from TAC-induced cardiac hypertrophy^40,41^, (ii) CTNNBIP1 (also known as ICAT), a protein that blocks activated beta-catenin from interacting with the transcription factor TCF/LEF, and deficiency of which causes heart enlargement^42^, (iii) HBP1, a negative regulator of TCF/LEF^43^ and (iv) GLIS2, a transcriptional repressor that binds to, and is cleaved by, p120 catenin^44^. We propose the beneficial effect exerted by antimiR-29 to result to a considerable extent from restoration of the inhibition of Wnt signaling. Formal proof for this hypothesis would require the inhibition of Wnt-signaling together with manipulation of miR-29 in cardiac myocytes in vivo, which should be the focus of further studies. Of note in this respect, the data presented here also support a previously suspected correlation between miR-29 and Wnt signaling in osteoclasts^45^.

An unexpected outcome of our studies in the pressure overload model was that antimiR-29 not only prevented cardiac hypertrophy, but also cardiac fibrosis. This is a remarkable finding, considering that elevation, rather than inhibition, of miR-29 suppresses fibrosis in tissues other than myocardium. Fibrosis—the secretion of extracellular matrix proteins such as collagens—occurs in response to various disease conditions. Based on target prediction algorithms, miR-29 has early been suspected to negatively regulate the expression of collagens and, indeed, this has been confirmed by manipulating miR-29 in vitro or in various tissues^13,30^. More importantly, studies on miR-29 in fibrosis disease models in liver, lung and kidney have demonstrated the potential benefit of elevating miR-29 levels^46–48^. In this respect, our findings that inhibition or genetic deficiency of miR-29 in vivo prevents (rather than promotes) myocardial fibrosis in a cardiac disease model were, at first, unexpected. We thus reasoned that the unique myocardial tissue context may determine the net balance of pro- or antifibrotic miR-29-controlled processes more than its individual activity in cardiac fibroblasts. The hypothesis of a dominant miR-29 function in CM finds support by our observations that (i) endogenous levels of miR-29 variants in vivo are substantially higher in CM, compared to CF (a discrepant previous observation in cultured CF^13^ seems attributable to aberrant expression that occurs during cultivation, see Supplementary Fig. 3d, e), that (ii) mice with CM-specific deficiency for the *miR-29b2/c* cluster (upon infection with iCre-encoding AAV9 vector) were largely protected from pressure overload-induced cardiac remodeling (consistently, AAV9-mediated overexpression exacerbated cardiac remodeling in this model) and that (iii) induction of fibrosis-involved collagens 1a1, 1a2 and 3a1 in TAC-exposed myocardium is reduced in miR-29-deficient or antimiR-29-treated mice (Figs 1f, 2g). Together, these data illustrate a function of miR-29 in the interplay between myocardial cell types that promote cardiac remodeling, and highlights a perspective for therapeutic inhibition of miR-29. Interestingly, inhibiting miR-29 may also prove beneficial in limiting aortic aneurysm progression^22^ and our data should prove valuable also with regard to the further development of this indication. Future studies will expand our understanding of which mechanisms are involved in miR-29 mode of action in cardiac fibroblasts. These studies should identify how miR-29 inhibition prevents cardiac fibrosis *in vivo* and determine the relevance of the Wnt signaling pathway in this anti-fibrotic effect. In light of the dynamic regulation of miR-29 observed after initiation of pressure overload in this study, the timing and duration of therapeutic miR-29 inhibition may warrant optimization as well as long term follow-up.

Whether the miR-29 family members exert individual roles in myocardium and to what extent there is functional redundancy among them has not been resolved yet. Given the reported nuclear localization of miR-29b^31,32^, it is well conceivable that this variant could have functions in addition to those of miR-29a and c. This may also explain (next to differing experimental conditions) why a previous study observed increased left ventricular fibrosis in TAC-treated, antimiR-29b-infused mice (compared to antimiR-control)^30^. Also, endogenous expression levels of each family member appear to vary, depending on experimental conditions and time points analyzed^13,23,30^. Thus, an even deeper comparison of miR-29 expression in cell types, organs and disease models may be rewarding in order to dissect individual functions of each variant.

The prohypertrophic function of miR-29 in CM and the profibrotic effect that comes with it provokes to ask whether global genetic deficiency of *miR-29* (*miR-29 ab1* ^*−/−*^ *b2c* ^*+/−*^ mice) or systemic antimiR-29 infusion induced fibrosis in organs such as kidney, liver or lung. We have not observed an increase of Sirius Red staining for extracellular matrix in *miR-29 ab1* ^*−/−*^ *b2c* ^*+/−*^ mice in these organs (Supplementary Fig. 2b). Nonetheless, to minimize the risk of unwanted side effects in these organs, miR-29-directed therapy against cardiac remodeling would likely benefit from targeted delivery to the myocardium and individual cell types therein - developments that are still in their infancy^49,50^. The unveiled unique hierarchy of miR-29 activities in different cardiac cell types in myocardium now provides a basis for such approaches.

## Methods

### Mouse models

Thoracic aortic constriction was performed on 8-week-old male C57BL/6 N mice (Charles River Laboratories), as described^51^ with small adaptations. Mice received buprenorphine (0.1 mg/kg s.c.) 60 min before intubation and anesthesia with isoflurane. Thoracotomy was performed between the second and third rib, and the aortic arch was narrowed by a ligature over a 27 G cannula. Until complete recovery from anesthesia, the mice remained in a warmed cage for 2–4 h under direct supervision. In sham surgery, only the chest was opened, but no ligation of the aorta was carried out. Cardiac dimensions and function were analyzed by pulse-wave Doppler echocardiography, before TAC/sham surgery and before the animals were euthanized. Then, mice were sacrificed to determine parameters of cardiac hypertrophy and fibrosis. The generation of *miR-29ab1* ^*−/−*^ mice has been described previously^28^. Hemizygous *miR-29b2/c* ^*fl/+*^ animals were generated as follows: for the targeting construct, two homologous arms of the ~ 10 kb *miR-29b-2/c* cluster KpnI fragment were isolated and amplified from 129/Ola genomic DNA and inserted in a diphtheria toxin-A (DT-A)-containing pUC18 vector^52^. 0.4 kb downstream of *miR-29c* (SphI) as well as upstream of *miR-29b-2* (NheI), two mutated loxP sites^53^ were inserted, the latter followed by a PGK-driven, FRT flanked hygromycin B cassette. After linearization (XmaI) and electroporation of the targeting construct to E14 129/Ola ES cells, clones resistant to hygromycin B (at 60 µg/ml) were screened by Southern blotting, followed by PCR and sequencing. Chimera obtained by morula aggregation were back-crossed at least 7 times in C57BL/6 background. Homozygous *miR-29b2/c* ^*fl/fl*^ were crossed with the *EIIa-Cre* line^54^ to produce full knockouts. All genotypes were verified by PCR analysis. For cardiotropic expression of exogenous miR-29, 5-week-old male wildtype mice received AAV9-miR-29a (2 × 10^12^ genome copies per mouse) or an AAV9 that encodes the nonrelated *C. elegans* miR-39 (as a negative control) by tail vein injection, and TAC was performed 3 weeks later. *miR-29 b2c* ^*fl/fl*^ and *miR-29 b2c* ^*+/+*^ neonatal mice were injected with AAV9-iCre as described previously^15^. Briefly, 3–4 day old mice were anesthetized by an injection of fentanyl (0.05 mg/kg), midazolam (5 mg/kg) and medetomidine (0.5 mg/kg). Under anesthesia the mice were injected 50 µl of the virus pericardially using a 30 G needle. Control antimiR and antimiR-29 (synthesized by Exiqon, see Fig. 2a for sequence) were administered intravenously at 20 mg/kg. Mice treated with PBS were also included as controls. Cardiac dimensions and function were analyzed by pulse-wave Doppler echocardiography before surgery and before the animals were euthanized. All animal studies were approved by the relevant authority (Regierung von Oberbayern, Munich, Germany) and performed in accordance with the relevant guidelines and regulations.

### Quantification of miR-29 in human myocardium

Informed consent was obtained from all subjects and the study was performed with the approval of the institutional ethical committees of the University of Würzburg, Germany, and the Hannover Medical School, Hannover, Germany. RNA from healthy human hearts was purchased from Biochain Institute. We analyzed cardiac tissue from patients undergoing aortic valve replacement because of aortic stenosis (*N* = 24, mean age 69.5 ± 18.19, male: female 16:8). For comparison, tissue of healthy human hearts was used (*N* = 23, mean age 38.61 ± 12.96, male: female 14:9). Cardiac biopsies were obtained from the left ventricle and frozen in liquid nitrogen. RNA was isolated using TriFast. Expression of miR-29a, b and c and RNU6B (normalization control) were measured by Taqman assays (ThermoFischer Scientific) as per manufacturer’s instructions.

### Isolation of cardiac myocytes and cardiac fibroblasts

Neonatal rat cardiac myocytes (NRCMs) and cardiac fibroblasts (NRCFs) were isolated from 0–1-day-old Sprague Dawley rats as described previously^26^. Briefly, the area close to the neck was disinfected and the rats were decapitated. The hearts were then excised and digested with collagenase type II (Worthington) and pancreatin (Sigma Aldrich) in CBFHH buffer (120 mM NaCl, 5 mM KCl, 0.8 mM MgSO_4_, 0.5 mM KH_2_PO_4_, 0.3 mM Na_2_HPO_4_, 20 mM HEPES, 5.6 mM Glucose, pH 7.3 including Penicillin/Streptomycin) at 37 °C. Every 10 min the solution containing the digested cells was transferred to a new tube containing FCS (Sigma Aldrich). The remaining not dissociated tissue residue was supplemented with fresh enzymatic solution for additional five rounds. After combining the cell suspensions, they were centrifuged at 50×*g* for 5 min and the pellet was resuspended in MEM medium containing 5% FCS, filtered (40 μm filter; BD), and pre-plated at 37 °C and 1% CO_2_ for 75 min onto 10 cm cell culture dishes (Nunc). Afterwards the supernatant containing the cardiac myocytes was collected, the cells were counted automatically (Countess Cell Counter, Invitrogen) and plated. Adult mouse cardiac myocytes (AMCMs) and cardiac fibroblasts (AMCFs) were isolated from wildtype C57BL/6 N mice. Briefly, hearts were excised and the aorta was cannulated and perfused with buffer A (in mM: 113 NaCl, 4.7 KCl, 0.6 KH_2_PO_4_, 0.6 Na_2_HPO4, 1.2 MgSO_4_, 12 NaHCO_3_, 10 KHCO_3_, 10 HEPES, 30 taurine). For dissociation of cells collagenase type II (Worthington) was included in the buffer. After dissociation the cell suspension was incubated for 10 min at 37 °C, allowing the cardiac myocytes to sediment. Afterwards the cardiac fibroblast-enriched supernatant and the pellet were resuspended in buffer B (47.5 ml perfusion buffer A, 2.5 ml FCS, 62.5 ml 10 μM CaCl_2_). Calcium reconstitution of cardiac myocytes was achieved by addition of increasing concentrations of CaCl_2_ to a final concentration of 100 µM. The cardiac myocyte fraction was seeded in MEM (5% FCS, 10 mM 2,3-butanedione monoxime, 2 mM L-glutamine and 1% penicillin/streptomycin) at 37 °C and 5% CO_2_. For cardiac fibroblast cultures, the supernatant was centrifuged for 5 min (400 x g), resuspended in 5% FCS MEM culture medium and plated on 6 cm culture dishes.

### Histochemical and immunohistochemical analyses

Fixation of tissues for histology was in 4% paraformaldehyde. Tissue slices(6 µm) were then processed for hematoxylin/eosin staining. To determine collagen deposition, Sirius Red and Fast Green staining was performed on paraffin sections (8 µm thick) of murine hearts. The percentage of Sirius Red positive area was used as a quantitative measure of collagen deposition. For determination of cardiac myocyte cross-sectional areas, 6 µm thick paraffin-embedded sections were prepared from murine hearts and subsequently stained with Alexa Fluor 647-conjugated wheat-germ agglutinin (WGA, Life Technologies, 1:200 dilution) for cell border determination and SYTOX Green (Life Technologies, 1:1000 dilution) for nuclei detection. Images were taken from areas of transversely cut muscle fibers by confocal microscopy. A Leica TCS SP5 II confocal microscope with a ×20 objective, laser lines 488 nm for SYTOX Green and 633 nm for WGA were used for image acquisition. The MetaMorph software (Molecular Devices) was programmed to recognize individual cells based on the WGA staining in an automated fashion. Proper thresholds were set for background and excessive fibrosis exclusion. MetaMorph’s integrated morphometry analysis tool was then used to calculate the average cell area of the cardiac myocytes.

### Quantitative real-time PCR

Total RNA was isolated using the RNeasy Mini kit (Qiagen) and Superscript II (Invitrogen) was used to retrotranscribe 500 ng of RNA following the manufacturer’s instructions. For quantitative real time PCR the FastStart universal SYBR Green Master Mix (Roche) was used. Target-specific primers were designed and their sequences are listed below (each gene symbol is followed by the forward and reverse primers. In total 2 µl template, 400 nM primers and 1× SYBR Green master mix were pippeted together and the qPCR was performed in a StepOnePlus Real-Time-PCR System (Applied Biosystems).

*Nppa* mouse (GCTTCCAGGCCATATTGGAG, GGGGGCATGACCTCATCTT);

*Myh6* mouse (GCCCAGTACCTCCGAAAGTC, GCCTTAACATACTCCTCCTTGTC);

*Myh7* mouse (ACTGTCAACACTAAGAGGGTCA, TTGGATGATTTGATCTTCCAGGG);

*Gsk3β* mouse (CAAGCAGACACTCCCTGTGA, AATGTCTCGATGGCAGATCC);

*Rcan1* mouse (GCTTGACTGAGAGAGCGAGTC, CCACACAAGCAATCAGGGAGC);

*Gapdh* mouse (GTGAAGGTCGGTGTGAACG, TCGTTGATGGCAACAATCTC);

*Col1a1* mouse (CTGGCAAGAAGGGAGATGA, CACCATCCAAACCACTGAAA);

*Col1a2* mouse (AGGTCTTCCTGGAGCTGATG, ACCCACAGGGCCTTCTTTAC);

*Col3a1* mouse (ACAGCAAATTCACTTACACAGTTC, CTCATTGCCTTGCGTGTTT).

### Quantification of miR-29 in isolated cells or tissue

Total RNA was prepared using TriFast (peqLab) and 10 ng were reverse-transcribed, using the Universal cDNA Synthesis Kit II (Exiqon). The cDNAs were quantified using the FastStart universal SYBR Green Master Mix (Roche), and modified primers for individual miR-29 variants (miRCURY LNA PCR primer sets) or for U6 snRNA (Exiqon) were used for qPCR quantification in a StepOnePlus Real-Time-PCR System (Applied Biosystems), with parameters recommended by Exiqon. For qPCR analysis, individual primer efficiencies were determined using a regression model (LinRegPCR software), and analyses were done using the Pfaffl method.

### Assessment of cardiac myocyte hypertrophy

NRCMs were plated onto optics-optimized 96-well plates (Ibidi, Martinsried, Germany) in MEM medium that contained 1% FCS. Twenty-four hours later, cells were transfected with miRNA mimics (Ambion) or inhibitors (Exiqon) at a final concentration of 50 nM, using Lipofectamine2000 (Invitrogen). After 6 h of transfection, the medium was exchanged to 0.1% FCS in MEM medium. Forty-eight hours later, the medium was exchanged to 0.1% FCS in MEM, supplemented with or without phenylephrine to 50 µM (PE; Sigma-Aldrich). After 96 h of transfection, cells were washed twice with PBS and fixed for 10 min with paraformaldehyde (4%). When IWR-1 (Sigma-Aldrich, 10 µM) or 11R-VIVIT (Calbiochem, 5 µM) was used, it was added to the medium 6 h after transfection. Immunostainings of NRCMs in 96-well format and automated cell size measurement were performed as described previously^26^. Cells were fixed using 4% paraformaldehyde (5 min at room temperature) and permeabilized with Triton-X (0.2% in PBS). The anti-α-actinin antibody (monoclonal, sarcomeric, clone EA-53, Sigma Aldrich) in a 1:1000 dilution was then incubated for 45 min at 37 °C, followed by incubation at 37 °C for 30 min with an Alexa-488-coupled antibody (1:200 dilution, Invitrogen) and 4′,6-diamidin-2-phenylindol (DAPI) (final concentration 1 µg/µl, Sigma Aldrich). Cardiac myocytes were assigned green, whereas non-myocytes are identified by red nuclei.

### Reporter activity assays

NFAT activity was detected by luminescence using a luciferase reporter construct containing NFAT binding sites (a kind gift from J. Molkentin, University of Cincinatti). In order to test miR-29 mimic efficiency in vitro, a complementary binding site for miR-29c was inserted into a pmiR-RL-TK2 vector (a kind gift from G. Meister, University of Regensburg), downstream of the sequence that encodes firefly luciferase. NRCMs were transfected with the reporter plasmids and a miR-control or miR-29a (final concentration 50 nM) using Lipofactamine2000 (Invitrogen) as recommended by the manufacturer. Firefly and renilla luciferase activities were determined using One-Glo or Dual-Glo luciferase assay kits (Promega) according to the manufacturer’s instructions. For NFAT activity assays, the level of luciferase activity was normalized to the total protein content.

Wnt activity was detected by luminescence using a luciferase reporter construct containing binding sites for TCF/LEF (M50 Super 8x TOPFlash, Addgene plasmid # 12456)^55^. A plasmid carrying mutated TCF/LEF binding sites upstream of a luciferase reporter was used as a control (M51 Super 8x FOPFlash, Addgene plasmid # 12457)^55^. NRCMs were transfected with the reporter plasmids and a miR-control or miR-29a (final concentration 50 nM) using Lipofectamine2000 (Life Technologies) as recommended by the manufacturer. Six hours before the end of the experiment Wnt3a (Cell Guidance Systems Ltd) was added to the medium at a final concentration of 50 ng/ml. Luciferase activity was determined using One-Glo luciferase assay kits (Promega) according to the manufacturer’s instructions.

In order to test if miR-29 directly targets *Gsk3β*, *Ctnnbip1*, *Hbp1* and *Glis2*, 430–1240 base pairs of their 3′ untranslated regions (3′ UTRs; including the miR-29 binding sites therein) were amplified by PCR and cloned into a double fluorophore-encoding plasmid. These plasmids contain the 3′-UTRs of the *Gsk3β*, *Ctnnbip1*, *Hbp1* and *Glis2* genes downstream of the eGFP gene under the control of the CMV promoter. As an internal control for transfection, the reporter construct also comprised the tdTomato gene under a second CMV promoter, and data were assessed as the ratio of both fluorescence intensities. Mutation of miR-29 binding site was carried out with primers carrying the nucleotide exchanges, using the Q5 site-directed mutagenesis kit (New England Biolabs). HEK293 cells were cultured in modified Eagle medium (MEM) with 5% fetal calf serum (FCS). Plasmids were co-transfected into HEK293 cells, using Lipofectamine2000 (Life Technologies) as recommended by the manufacturer, with miR-ctrl or miR-29a (5 nM, Ambion) in a 96 well plate (Ibidi) and fluorescence intensities measured 48 h later.

### Construction and production of AAV9 vectors

A genomic fragment containing 700 nucleotides of the miR-29a precursor was amplified by PCR from mouse genomic DNA and cloned into a self-complementary AAV backbone plasmid. The *C. elegans* miR-39 (350 nucleotides) was synthesized, inserted in the AAV backbone plasmid and used as a control. The open reading frame of codon-improved Cre-recombinase (iCre) was subcloned into a predigested self-complementary AAV vector (scAAV-CMV).

HEK293-T cells were grown in triple flasks for 24 h (DMEM, 10% fetal bovine serum) before transfection. The transgene plasmid and the helper plasmid (pDP9rs, kindly provided by Roger Hajjar, Icahn School of Medicine at Mount Sinai, New York) were transfected into the HEK293 cells using Polyethylenimine (Sigma-Aldrich). After 72 h, the viruses were purified from benzonase-treated cell crude lysates over an iodixanol density gradient (Optiprep, Sigma-Aldrich). AAV titers were determined by a real-time polymerase chain reaction (PCR) on vector genomes using the SYBR Green Master Mix (Roche).

### Secretome analysis

Neonatal rat cardiac myocytes (NRCM) were isolated and seeded in a 6 well plate. One day later, NRCMs were transfected with LNA-modified antimiR-29 or a control molecule (both from Exiqon) at a final concentration of 50 nM, using Lipofectamine2000 (Life Technologies). After 6 h of transfection, the medium was exchanged to 0.1% FCS in MEM medium. After 48 h, the conditioned medium was collected and centrifuged at 3000 g for 10 min to remove cell debris. The supernatant was transferred into a new tube and stored at −80 °C. Conditioned media (2 ml each) were concentrated into 100 μl using 3 kD MWCO spin column and 30 μl was mixed with 4× Laemmli buffer and proteins were separated by 4–12% Bis-Tris gel at 130 V for 1.5 h. The gels were silver stained and each lane was cut into 12 bands without any gap. The gel bands were digested overnight on a ProGest robotic digestor. The digested peptides were lyophilized and resuspended in 20 μl of 2% ACN, 0.05% TFA. Then 15 μl of peptides were separated by reversed phase nano-flow HPLC (UltiMate 3000 RSLCnano system with PepMap C18 column, 25cm x 75 μm) (LC gradient: 0–5 min, 2–10%B; 5–65 min, 10–30%B; 65–70 min, 30–40%B; 70–80 min, 99%B; 80–100 min, 2%B; A = 0.1% FA in HPLC H_2_O; B = 80% ACN, 0.1% FA in HPLC H_2_O) and directly analyzed by LTQ Orbitrap XL using full ion scan mode m/z range 350–1600 with Orbitrap, resolution 60000 (at m/z 400), lock mass m/z = 445.12003. MS/MS was performed using CID on the top six ions with dynamic exclusion for 120 s.

Raw files were searched against UniProt/SwissProt rat database (2014_01, 7894 protein entries), using Mascot 2.3.01. The mass tolerance was set at 10 ppm for the precursor ions and at 0.8 Da for fragment ions. Carboxyamidomethylation of cysteine was used as a fixed modification and oxidation of methionine, proline and lysine as variable modifications. Trypsin was used as enzyme and 2 missed cleavages were allowed. Search results were loaded into Scaffold (version 4.3.2) and the following filters were used: peptides probability > 95%, protein probability > 99%, minimum 2 unique peptides identified. The normalized spectrum count was used for further analysis.

### Statistics

The GraphPad Prism software (version 6) was used for all statistical tests. The distribution of the data was determined by Shapiro Wilk’s or Kolmogorov-Smirnov test for normality. F-test or Bartlett’s test was used to test for common variance. All quantitative data are reported as means ± SEM. The Student’s *t*-test assessed differences between two means. If multiple means had to be assessed, one-way or two-way ANOVA followed by Bonferroni’s post hoc test were performed. If the *n* number was not sufficient for normality testing, non-parametric tests (Mann–Whitney U-test or one-way ANOVA followed by Dunn’s test analysis) were used. Any *P* value less than 0.05 was considered to be significant and denoted as * for *P* < 0.05, ** for *P* < 0.01 and *** for *P* < 0.001. Not significant differences are abbreviated as “N.S.”.

### Data availability

All data generated or analyzed during this study are presented in this article and its Supplementary Information File, or are available from the corresponding author upon reasonable request.

## Electronic supplementary material

Supplementary Information
