# Supplementary material for: Cardiac myocyte miR-29 promotes pathological remodeling of the heart by activating Wnt signaling
Source: Nat Commun. 2017 Nov 20;8:1614. doi: 10.1038/s41467-017-01737-4 (PMC5696364; doi:10.1038/s41467-017-01737-4)
Supplement: Supplementary file 1 — Supplementary Information [file 41467_2017_1737_MOESM1_ESM.pdf]

Supplementary Information

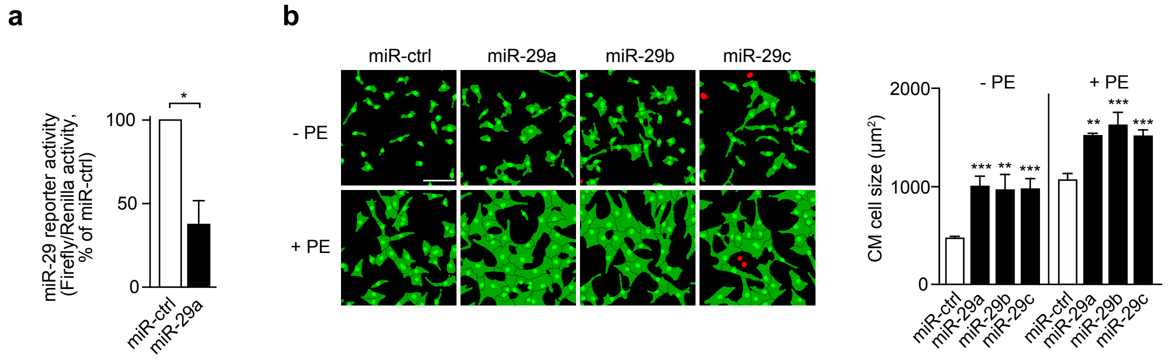

Supplementary Figure 1.
Each miR-29 family member promotes hypertrophy of cardiac myocytes in vitro.
(a) Effect of a synthetic mimic of miR-29a on reporter suppression in primary cardiac myocytes. MiR-29a activity was determined by a reporter system where luciferase is under control of a miR-29a binding site, located 3' to the coding sequence of luciferase. Cardiac myocytes from neonatal rat hearts (NRCMs) were transfected with miR-29a or miR-ctrl (50 nM each). Data are from 4 independent experiments performed in triplicate. P values determined by Student's *t*-test. (b) (Left) Automated cell detection-based imaging to determine cardiac myocyte cell size and purity of the cell preparations. After transfection of synthetic miRNAs or a control molecule, NRCMs were treated with the prohypertrophic α1-adrenoceptor agonist phenylephrine (50 μM for 48 h), fixed and stained with an antibody directed against α-actinin and DAPI. Automated cell scoring was then employed to determine cardiac myocyte dimensions (green) and the presence of contaminating non-myocytes (α-actinin-negative, depicted in red). Scale bar: 100 μm. (Right) Quantitative analysis of the results. Data are from 5-10 independent primary cell isolations. P values were determined by one-way ANOVA followed by Bonferroni's post hoc test. All quantitative data are reported as means ± SEM. *P<0.05, **P<0.01, ***P<0.001.

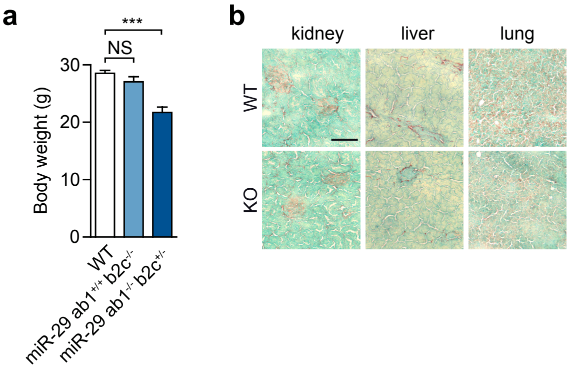

Supplementary Figure 2.

Body weight and Sirius Red staining for kidney, liver and lung of *miR-29* deficient mice.

(a) Body weight of 3 months-old wildtype mice and of mice with miR-29 ab1$^{+/+}$ b2c$^{-/-}$ and miR-29 ab1$^{-/-}$ b2c$^{+/-}$ genotype, respectively. Data are from 7-11 mice per group. ***P<0.001 determined by one-way ANOVA followed by Bonferroni's post hoc test; NS: not significant. (b) Representative image sections from Sirius Red/Fast Green-stained organs after TAC. WT: wildtype, KO: miR-29 ab1$^{-/-}$ b2c$^{+/-}$. Scale bar: 100 µm. All quantitative data are reported as means ± SEM.

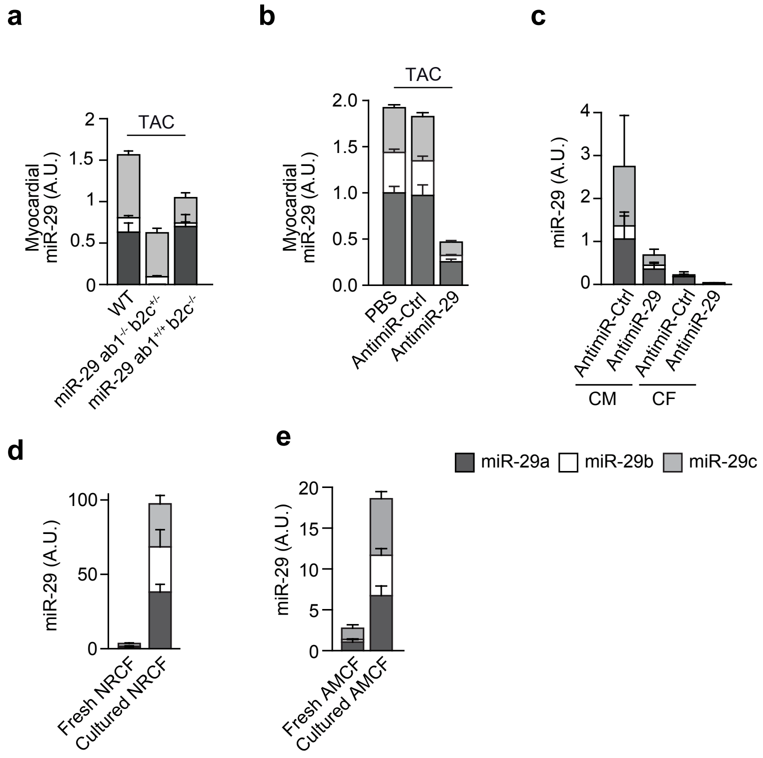

Supplementary Figure 3.

Deregulation of miR-29 upon cultivation of primary cells and efficacy of miR-29 inhibition in vivo.

(a-e) Determination of miR-29a, b and c by real time PCR in RNA preparations from primary cardiac cells or left ventricular myocardium. (a,b) MiR-29 levels in left myocardium from (a) miR-29-deficient mice (n = 3-5 mice/group) and (b) antimiR-treated mice three weeks after TAC (n = 6-9 mice/group). (c) Determination of miR-29 in primary cardiac myocytes and fibroblasts isolated from the hearts of mice two days after antimiR-29 or antimiR-Ctrl treatment; n = 4 mice per group. (d,e) MiR-29 levels in (d) neonatal rat cardiac fibroblasts and (e) adult mouse cardiac fibroblasts directly after isolation and after one to two weeks of culture; n= 4-7 independents cell preparations. All quantitative data are reported as means ± SEM.

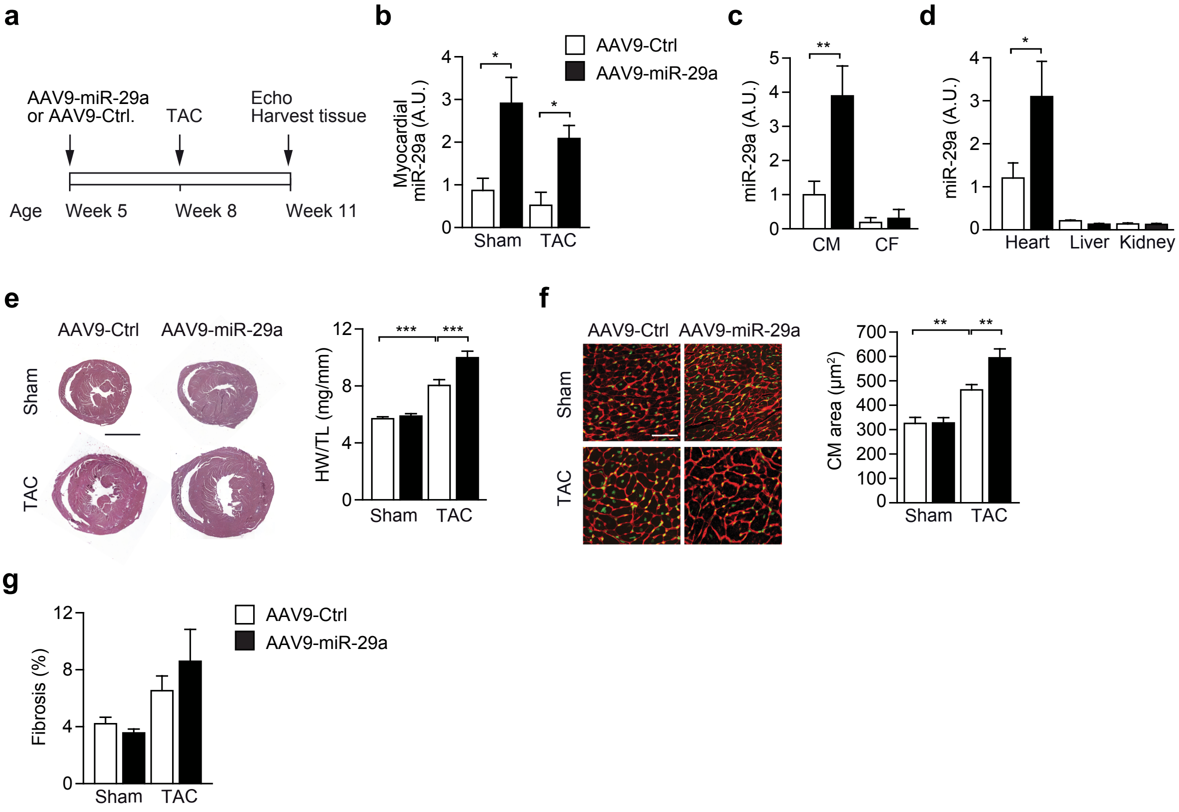

Supplementary Figure 4.

Cardiac myocyte-targeted expression of miR-29 using adeno-associated virus serotype 9 (AAV9).

(a) Design of the study. 2 $x10^{12}$ viral particles (AAV9-miR-29a or AAV9-control (AAV9-cel-miR-39)) were delivered to 5 weeks old wildtype mice via tail vein injection. Three weeks later, mice were subjected to TAC or sham surgery. At week 11, mice were examined by echocardiographic analysis and sacrificed immediately afterwards. (b) MiR-29a expression level in cardiac tissue from mice treated as in (a); n = 4-7 mice per group. (c) MiR-29a level in cardiac myocytes and cardiac fibroblasts isolated from mice treated as in (a); n= 4-6 mice per group. (d) MiR-29a level in heart, liver and kidney from WT mice injected with AAV9-miR-29a or AAV9-Ctrl; n = 4-6 mice per group. (e) (Left) Hematoxylin eosin staining of mid-ventricular sections; scale bar: 2 mm. (Right) Heart weight-to-tibia length ratio; n = 8-10 mice per group. (f) (Left) WGA staining of left ventricular myocardium treated as in (a); scale bar: 50 µm. (Right) Quantitative analysis of the results; n = 6-9 mice per group. (g) Quantitative analysis of left ventricular fibrosis; n = 7-10 mice per group. All quantitative data are reported as means ± SEM. $^{*}P<0.05$, $^{**}P<0.01$, $^{***}P<0.001$ as determined by Student's t-test (b) or two-way ANOVA followed by Bonferroni's post hoc test (c-f).

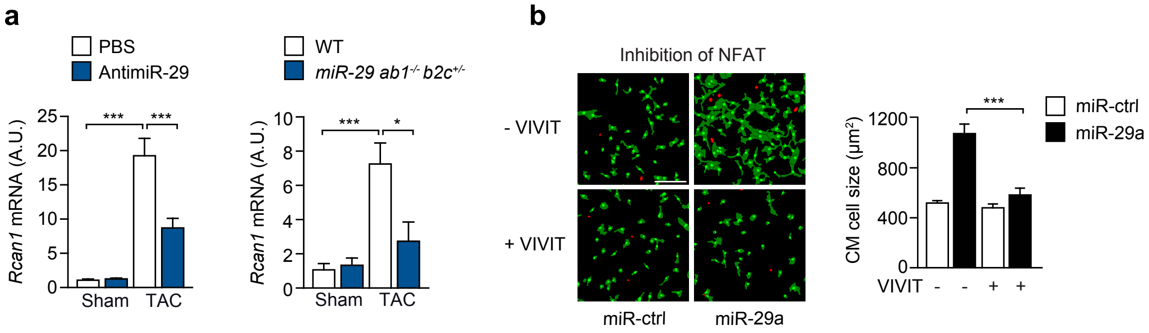

Supplementary Figure 5.

MiR-29 promotes NFAT-mediated hypertrophy of cardiac myocytes.

(a) Inhibition or deficiency of miR-29 reduces NFAT-mediated induction of Rcan1. 8 weeks-old wildtype mice (left) or mice with *miR-29 ab1*$^{-/-}$ *b2c*$^{+/-}$ genotype (right) were subjected to sham or TAC surgery. In the case of antimiR-29 or PBS, consecutive injections with 20 mg/kg were carried out on days 1-3, as illustrated in Fig. 2a and as described in Methods. At week 11, RNA was isolated from left ventricular tissue, and Rcan1 mRNA was quantified by qPCR analysis; n = 5-9 mice/group. (b) The NFAT-inhibitor VIVIT prevents miR-29 from inducing cardiac myocyte hypertrophy. (Left) Representative image segmentations of NRCM after transfection with synthetic miR-29a (or miR-ctrl) and addition of 11R-VIVIT (5 µM for 48 h); scale bar: 100 µm. (Right) Quantitative analysis of the results. Data are from 6 independent experiments each performed in triplicate. All quantitative data are reported as means ± SEM. *P<0.05, ***P<0.001 determined by two-way ANOVA followed by Bonferroni's post hoc test.

Supplementary Table 1.
Echocardiography parameters obtained from the study depicted in Fig. 1

Treatment	Sham	Sham	Sham	TAC	TAC	TAC
Genotype	WT	*miR-29ab1-/- b2c+/-*	*miR-29ab1+/+ b2c-/-*	WT	*miR-29ab1-/- b2c+/-*	*miR-29ab1+/+ b2c-/-*
N number	9	5	6	6	4	5
LVID;d (mm)	4.03 ± 0.08	3.63 ± 0.13	3.99 ± 0.02	4.69 ± 0.14##	3.66 ± 0.16 **	3.89 ± 0.17 *
LVID;s (mm)	2.91 ± 0.12	2.64 ± 0.10	3.12 ± 0.09	3.81 ± 0.13##	2.84 ± 0.16 **	2.87 ± 0.27 **
LV Vol;d (ml)	71.44 ± 3.24	60.28 ± 3.21	67.86 ± 1.31	103.27 ± 6.69##	62.48 ± 6.05 **	66.44 ± 7.52 *
LV Vol;s (ml)	33.35 ± 3.33	30.33 ± 1.55	37.64 ± 2.26	63.08 ± 4.72##	33.44 ± 4.84 **	33.35 ± 8.40
EF (%)	53.99 ± 3.09	56.23 ± 2.91	52.48 ± 3.48	37.93 ± 1.97##	46.91 ± 3.27	52.09 ± 6.31 *
FS (%)	28.87 ± 1.47	28.39 ± 2.19	32.89 ± 1.09	18.88 ± 0.76###	23.63 ± 1.62 *	26.82 ± 3.95 *

LV internal diastolic diameter (LVID;d), LV internal systolic diameter (LVID;s), LV diastolic volume (LV Vol;d), LV systolic volume (LV Vol;s), LV ejection fraction % (EF(%)), LV fractional shortening % (FS(%)). ##P<0.01, ###P<0.001 versus Sham WT, *P<0.05 and **<0.01 versus TAC WT. P values were calculated using unpaired, two-tailed Student's *t*-tests. Data represent means ± SEM.

Supplementary Table 2.
Echocardiography parameters obtained from the study depicted in Fig. 2

Treatment	Sham PBS	Sham LNA-ctrl	Sham LNA-29	TAC PBS	TAC LNA-ctrl	TAC LNA-29
Genotype	WT	WT	WT	WT	WT	WT
N number	14	5	5	8	5	6
LVID;d (mm)	4.16 ± 0.12	4.07 ± 0.11	4.06 ± 0.14	4.25 ± 0.10	4.63 ± 0.22 #	4.07 ± 0.08 *
LVID;s (mm)	3.18 ± 0.11	3.01 ± 0.16	3.08 ± 0.10	3.71 ± 0.09	4.17 ± 0.23 ##	3.24 ± 0.15 **
LV Vol;d (ml)	78.19 ± 4.97	73.62 ± 4.82	73.35 ± 6.01	81.72 ± 4.97	101.52 ± 11.26#	73.30 ± 3.29 *
LV Vol;s (ml)	41.58 ± 3.28	35.96 ± 4.56	37.83 ± 2.92	59.31 ± 3.75	79.58 ± 10.69##	43.11 ± 5.11 **
EF (%)	46.93 ± 2.32	52.03 ± 3.26	48.36 ± 1.72	27.34 ± 2.42	22.35 ± 2.03###	41.90 ± 4.73 **
FS (%)	23.48 ± 1.46	26.49 ± 2.03	24.15 ± 1.06	12.67 ± 1.25	10.20 ± 0.95###	20.56 ± 2.59 **

#P<0.05, ##P<0.01, ###P<0.001 versus Sham LNA-ctrl, *P<0.05 and **P<0.01 versus TAC LNA-ctrl. P values were calculated using unpaired, two-tailed Student's *t*-tests. Data represent means ± SEM.

Supplementary Table 3.
Echocardiography parameters obtained from the study depicted in Fig. 4

Treatment	Sham	Sham	TAC	TAC
Genotype	*miR-29b2c+/+*	*miR-29b2cfl/fl*	*miR-29b2c+/+*	*miR-29b2cfl/fl*
N number	8	4	6	4
LVID;d (mm)	3.85 ± 0.06	4.18 ± 0.22	4.58 ± 0.20 #	4.24 ± 0.13
LVID;s (mm)	2.71 ± 0.09	3.03 ± 0.33	4.13 ± 0.21 ###	3.50 ± 0.04 **
LV Vol;d (ml)	64.19 ± 2.48	78.72 ± 10.12	97.9 ± 9.96 #	80.78 ± 5.49
LV Vol;s (ml)	27.65 ± 2.23	38.02 ± 10.93	78.12 ± 8.85 #	50.84 ± 1.6 **
EF (%)	57.27 ± 2.13	54.12 ± 6.73	22.25 ± 2.06###	38.40 ± 4.26 *
FS (%)	29.72 ± 1.36	28.13 ± 3.99	10.99 ± 1.23###	21.59 ± 0.59 **

#P<0.05, ###P<0.001 versus Sham *miR-29b2c+/+*, *P<0.05 and **P<0.01 versus TAC *miR-29 b2c+/+*. P values were calculated using unpaired, two-tailed Student's *t*-tests. Data represent means ± SEM.

Supplementary Table 4. Echocardiography parameters obtained from the study depicted in Supplementary Fig. 4

Treatment	Sham AAV-Ctrl	Sham AAV-29	TAC AAV-Ctrl	TAC AAV-29
Genotype	WT	WT	WT	WT
N number	9	7	7	6
LVID;d (mm)	4.12 ± 0.07	4.01 ± 0.07	4.21 ± 0.12	4.67 ± 0.16 *
LVID;s (mm)	3.12 ± 0.07	2.96 ± 0.04	3.43 ± 0.13 #	4.05 ± 0.16 *
LV Vol;d (ml)	75.35 ± 3.16	70.48 ± 2.77	79.64 ± 5.21	102.14 ± 7.89 *
LV Vol;s (ml)	38.84 ± 2.14	33.96 ± 1.12	49.09 ± 4.11 #	73.22 ± 6.89 *
EF (%)	48.45 ± 1.74	51.62 ± 1.53	38.61 ± 2.98 ##	28.26 ± 3.21 *
FS (%)	24.21 ± 1.05	26.08 ± 0.97	18.62 ± 1.60 ##	13.25 ± 1.63 *

#P<0.05, ##P<0.01 versus Sham AAV-ctrl, *P<0.05 versus TAC AAV-ctrl. P values were calculated using unpaired, two-tailed Student's *t*-tests. Data represent means ± SEM.
